# Quality Evaluation and Browning Control in the Multi-Stage Processing of *Mume Fructus* (Wumei)

**DOI:** 10.3390/foods13020272

**Published:** 2024-01-15

**Authors:** Lei Gao, Hui Zhang, Hui Wang, Ai-Chao Li, Min Wu, Qing-Zhu Wang, Zhi-An Zheng

**Affiliations:** 1 College of Engineering, China Agricultural University, Beijing 100083, China; 2 College of Food Science and Technology, Nanjing Agricultural University, Nanjing 210095, China

**Keywords:** *Mume Fructus*, processing strategy, browning reaction, temperature and humidity control, quality evaluation

## Abstract

The dried *Mume Fructus* (MF), called Wumei in China, is a unique food with medicinal and edible effects. But its actual production method is outdated with low efficiency and inconsistent quality. This study systematically investigated the influence of moisture content (MC), temperature, and relative humidity (RH) on the browning reaction and quality characteristics of the MF and proposed a continuous processing strategy of the three-stage variable process for MF production based on the precise process control of the temperature and the RH. The production process of MF was divided into three stages: preliminary dehydration, browning, and drying. The results showed that the browning reaction rate and drying efficiency were optimal when the MC of the raw materials was reduced to 50%. In the browning stage, the degree of browning was better, and the antioxidant capacity reached the maximum of 64.38 mg/g DM under a processing temperature of 80 °C and an RH of more than 60%. As the RH increased, the drying rate decreased, and the ash content exhibited an increase. Therefore, the optimal processing parameters for the browning stage were determined to be a temperature of 80 °C and an RH of 60%. In the final drying stage, a temperature of 60 °C coupled with a dehumidification mode proved sufficient to ensure efficient drying without compromising the quality of the MF. This study revealed the reaction mechanism of the rapid browning processing of MF, which has important guiding significance for the rapid processing of browning foods.

## 1. Introduction

*Mume Fructus* (MF) is a kind of specially processed product derived from the nearly ripe fruit of the *Prunus Mume* (PM) Sieb. et Zucc., a plant that belongs to the Rosaceae family [1]. The PM tree is mainly cultivated in China, Japan, South Korea, North Korea, and Southeast Asian countries. The PM is known as plum, Qingmei, or sour plum in China, Japanese apricot or MF in Japan, and maesil or oumae in Korea [2,3]. The acidity of PM ranges from 4.62% to 6.78%, including citric acid, malic acid, succinic acid, and so on. It has strong antibacterial and antiseptic properties. PM also contains abundant flavonoids, polyphenols, and other bioactive substances, giving it strong antioxidant capabilities [3,4]. As a unique fresh fruit and easily accessible food ingredient, PM has diverse applications, including the production of plum juice, plum wine, preserved plums, and medicinal MF [5]. MF is commonly used as a traditional Chinese medicine with a long history, marked by its high medicinal and nutritional value. It is rich in organic acids, amino acids, polysaccharides, flavonoids, polyphenols, phytosterols, and other components. In the 2020 Chinese Pharmacopoeia, citric acid content was adopted as an important quality control parameter for MF. It has been observed to have positive effects in relieving and treating symptoms such as lung Qi deficiency, chronic cough due to Yin deficiency, prolonged diarrhea, and excessive thirst due to Yin deficiency. Modern pharmacological research has shown that MF contains multiple active ingredients with antibacterial, anti-inflammatory, anti-tumor, anti-ulcer, antiviral, antioxidant, and anti-fertility activities [1,6,7].

**Figure 1:**
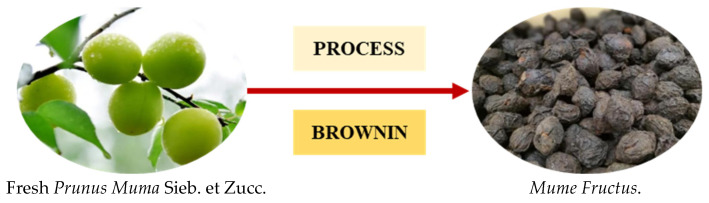
Contrast diagram of PM and MF.

Fresh PM is a seasonal produce with a moisture content ranging from 80% and 90%. It is prone to decay and spoilage after harvesting and has a relatively brief storage period [8,9]. As shown in Figure 1, PM is usually smoked to become MF in production areas of China. The specific process is to place yellow PM, which is nearly mature, on a breathable kang, and then the fruits are covered with a layer of red bamboo mat. At the same time, half-dried miscellaneous wood roots or withered PM tree roots are burned below, so the heat and smoke are used to smoke the PM on the kang. During the smoking process, it is necessary to flip the PM at regular intervals to allow it to be evenly dehydrated. In the middle and later stages of processing, it is necessary to select MF with suitable dryness [10]. According to the 2020 Chinese Pharmacopoeia and market evaluations of the quality of MF, aside from its size, MF with a darker coloration (black or brown) is usually considered to be of higher quality [11]. However, the mechanism and regulation mechanism of browning during the processing of MF are unclear, which limits the production of standardized MF on a large scale to some extent. Furthermore, traditional smoking processing also has problems such as a long processing time (5–7 days), low processing efficiency, high labor intensity, uneven drying, and difficulty in ensuring quality.

The browning reaction comprises two types: enzymatic browning (EB) and non-enzymatic browning (NEB). EB primarily arises from the oxidation reaction of organic substances catalyzed by enzymes, leading to the formation of a series of intermediate products and eventually forming brown compounds. In contrast, NEB is a browning reaction occurring without enzymatic catalysis, involving oxygen and organic matter [12,13]. During the processing of MF, NEB is the predominant type [10]. Many factors can affect NEB, including the concentration of reactants, pH, temperature, water activity, humidity, and time [14,15,16]. Therefore, some researchers have adjusted the temperature and humidity conditions during processing to promote the rate and progression of NEB. Ai et al. investigated the influence of high-temperature and high-humidity hot air impingement drying on the drying characteristics, weight loss, color, microstructure, and active components of *Cistanche deserticola*. They observed that the appearance of *Cistanche deserticola* was dark and glossy, and the content of echinacoside and acteoside reached the maximum value when steamed at 95 °C and 60% RH for 20 min, 75 °C and 70% RH for 20 min, and 75 °C and 60% RH for 30 min [17]. Sun et al. compared the effects of different temperatures (65 °C, 75 °C, and 85 °C) and relative humidity (70%, 75%, 80%, and 85%) on the quality of processed black garlic. They found that humidity and temperature significantly affected the content of various nutrients in black garlic, and the best color and quality were obtained when processed under conditions of 75 °C and 85% RH [18]. In addition, some researchers also achieved the browning processing of *Polygonatum sibiricum* Red through the nine-steaming and nine-processing method [19], in which steaming utilized higher temperature and humidity.

As a food and medicine with abundant nutrition and great medicinal value, MF enjoys widespread popularity. Appropriate processing techniques can not only improve its product quality and enhance its medicinal effects but also shorten processing time and reduce energy consumption. However, to the best of our knowledge, there are currently no reports on the impact of drying kinetics and quality attributes of MF using a precise control drying method based on temperature and humidity, nor are there any reports on the mechanism involved. Therefore, the main objectives of this study are (1) to establish the correlation between moisture content and water activity during the processing of PM into MF and determine the optimal starting point and corresponding water activity for the rapid browning reaction (RBR) in the multi-stage continuous drying process and (2) to investigate the effects of different temperatures and humidity conditions on the browning characteristics, drying characteristics, quality properties (total ash, extractives, citric acid, total flavonoids, and total phenols), and total antioxidant capacity of MF and propose an optimal processing scheme for MF processing. This work will be helpful in identifying suitable drying methods and optimal processing conditions for the mechanized processing of PM into MF and providing a theoretical basis for achieving the clean, efficient, and environmentally friendly processing of MF.

## 2. Materials and Methods

### 2.1. Raw Materials

Fresh PM (the Qingzhu variety) was picked from the Qingmei Orchard in Taiping Town, Zhao’an County, Zhangzhou City, Fujian Province. The fruits were stored in a refrigerator (4 ± 1 °C, 90% RH) to avoid cold damage and degradation of the active ingredients of fresh PM, and the storage time did not exceed 7 days. To ensure the uniformity of the test materials, fresh PM with no mechanical damage, hard texture, and uniform quality (diameter 3.3 ± 0.2 cm) was selected. Before processing, the fresh PM was taken out from the refrigerator and left at room temperature for about 30 min to balance the temperature difference between the inside and outside of the fruit with the environment. Then, the fruits were rinsed 2–3 times with clean tap water, and a toothpick was used to remove any remaining stems on the fresh PM. After rinsing, the surface moisture of the PM was immediately absorbed by using an absorbent paper. The PM was then placed in a dry tray located in a ventilated area, waiting for further processing. The moisture content of the fresh samples was determined using the AOAC test method [20]. The surface of fresh PM was evenly cut with a blade, and PM was dried with a vacuum dryer (D27-6050, Jinghong Instrument Co., Ltd., Shanghai, China) at 70 °C for 24 h. After three measurements, the moisture content of the fresh PM was determined to be 85.89 ± 0.8%.

### 2.2. Processing Equipment

**Figure 2:**
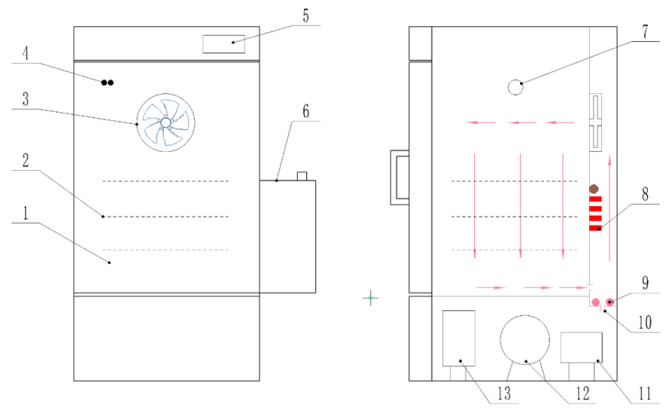
Structure diagram of hot-air drying equipment based on precise temperature and humidity control. 1. Drying chamber, 2. material rack, 3. circulating fan, 4. temperature and humidity sensor, 5. touch screen, 6. water tank, 7. test hole, 8. finned heater, 9. steam heating pipe, 10. water inlet, 11. liquid reservoir, 12. compressor, and 13. condenser.

This study conducted processing experiments on fresh PM by using three pieces of hot-air drying equipment with the same specification and precise control of temperature and humidity (THPC-HAD) (ICTHI-150, Instrument Equipment Co., Ltd., Shanghai, China). The schematic diagram of the THPC-HAD equipment is shown in Figure 2, and it was located at the College of Engineering, China Agricultural University. The THPC-HAD dryer was mainly composed of an internal circulation fan (turbulence fan), heating tubes, humidifying and dehumidifying devices, temperature and humidity sensors, compressors, and a control system [21]. In order to achieve the predetermined temperature and humidity process parameters, the dryer was required to be operated and preheated for 15 min before starting the drying experiment. After the equipment was turned on, a fin-type heater first heated the air inside the drying chamber, and then the internal circulation fan quickly and uniformly raised the temperature to the target temperature. Then, the control system directed the steam heating tubes to rapidly increase the humidity inside the drying chamber. Finally, through real-time monitoring and feedback from the temperature and humidity sensors, the control system commanded the humidifying and dehumidifying devices inside the drying chamber to achieve the set target relative humidity and maintain dynamic balance. The temperature control accuracy of this equipment was ±0.1 °C, and the humidity control accuracy was ±1.5% RH.

### 2.3. Determination of Optimal Moisture Transition Point for THPC-HAD

About 500 g of fresh PM samples was washed thoroughly and air-dried, and then we evenly spread them on a stainless steel tray lined with food-grade silicone mat and placed all of them in the THPC-HAD equipment oven for dehydration processing. The purpose was to investigate the changes in color and water activity under different moisture content states. During processing, the samples were taken out from the drying oven for data measurement every 2 h in the initial stage and every 1 h in the later stage until all of the moisture was removed. The recorded data included the changes in sample weight, color, and water activity. Each sample was measured at least 3 times. The dry basis moisture content was calculated based on the changes in sample weight (refer to Section 2.5). Simultaneously, relying on alterations in color, the total color difference, chromaticity, and browning index (*BI*) of the samples were computed (refer to Section 2.6) to determine the occurrence of the browning reaction during processing. In addition, the water activity of the samples was directly measured using a water activity meter (Aqualab 4TE, Decagon Company, Pullman, WA, USA) [22].

### 2.4. Multi-Stage Successive Combined Drying Strategy for Mume Fructus

This work conducted continuous processing experiments on PM utilizing THPC-HAD equipment with a three-stage variable process parameter. According to previous research and preliminary experimental results [23], the selected temperature range was 60–80 °C, and the selected relative humidity range was 40–80%. After preheating the THPC-HAD equipment to the set temperature and humidity parameters, prepared fresh PM samples were uniformly placed on three stainless steel trays covered with food-grade silicone pads, and then we put them on the tray rack in the drying chamber. The weight of PM in each tray was 500 ± 5 g. In the first stage, the focus was on the rapid drying and dehydration of fresh PM. The temperature parameter was set to 80 °C, and the relative humidity parameter was set to 40%. The objective was to reach the moisture transition point of the material on a dry basis (based on the results of the “determination of optimal moisture transition point by THPC-HAD and then processing with variable process parameters”). In the second stage, the main objective was to investigate the effect of different temperature and relative humidity conditions on the browning characteristics and quality of dried PM. It involved a coupling of the browning reaction, internal component transformation, drying, and dehydration. The temperature parameters were set to 60 °C, 70 °C, and 80 °C, while the relative humidity parameters were set to 40%, 60%, and 80%. The drying stages corresponding to different temperature and humidity conditions were labeled as I_1_ (60 °C, 40% RH), I_2_ (60 °C, 60% RH), I_3_ (60 °C, 80% RH), II_1_ (70 °C, 40% RH), II_2_ (70 °C, 60% RH), II_3_ (70 °C, 80% RH), III_1_ (80 °C, 40% RH), III_2_ (80 °C, 60% RH), and III_3_ (80 °C, 80% RH). The processing duration was 24 h. During the third stage, the focus was on drying and dehydration. The temperature was set to 60 °C, employing a continuous dehumidification mode until the material reached a safe moisture content of 16% as stipulated by the 2020 Chinese Pharmacopoeia [1]. The weight of the samples was recorded every 2 h in the first stage, every 4 h in the second stage, and every 2 h in the third stage.

### 2.5. Moisture Ratio (MR)

The moisture ratio (*MR*) during the process of processing PM into MF was calculated by using Formula (1) [24]:

(1) $$MR=\frac{M_{t}-M_{e}}{M_{0}-M_{e}}$$

where *M*_0_ is the initial moisture content of the PM sample, g/g (dry basis), and *M_t_* and *M_e_* are the dry basis moisture contents of PM at the time of processing *t* and at the time of processing to equilibrium, g/g (dry basis).

Due to the fact that the equilibrium dry base moisture content *M_e_* of PM was much lower than that of *M*_0_ and *M_t_*, the formula was simplified as follows [24]:

(2) $$MR=\frac{M_{t}}{M_{0}}$$

The moisture content of PM on a wet basis was calculated by using the formula [25]:

(3) $$M_{T}=\frac{W_{t}-G}{W_{t}}$$

where *M_T_* is the moisture content of PM on a wet basis, g/g; *W_t_* is the total mass of PM processing at time *t*, *g*; and *G* is the mass of dry matter in PM, g.

### 2.6. Image and Color Measurement

We utilized a colorimeter (Shengmingyang Co., Beijing, China) for the measurement of the colorimetric values (L*, a*, and b*) for both fresh PM and processed MF. At least 6 samples of PM/MF were selected under each processing condition, and each sample was measured 3 times repeatedly. The color of fresh PM and MF was measured using the CIE L*a*b* system, and the total color difference (Δ*E*) was calculated as follows [26]:

(4) $$\Delta E=\sqrt{{\left(L_{0}^{*}-L^{*}\right)}^{2}+{\left(a_{0}^{*}-a^{*}\right)}^{2}+{\left(b_{0}^{*}-b^{*}\right)}^{2}}$$

where $L_{0}^{*},a_{0}^{*}$, and $b_{0}^{*}$ are the color parameters of fresh PM and L*, a*, and b* are the color parameters of processed MF.

The chromaticity (*C*^0^) of the material was calculated according to the following formula:

(5) $$C^{0}={\left(a^{*2}+b^{*2}\right)}^{\frac{1}{2}}$$

The *BI* of the material was calculated according to the following formula:

(6) $$BI=100\times \left(\frac{X-0.31}{0.17}\right)$$

among

(7) $$X=\frac{\left(a^{*}+1.75L^{*}\right)}{\left(5.645L^{*}+a^{*}-3.012b^{*}\right)}$$

The color of both fresh PM and MF was determined using a computer vision system (CVS) placed in the Laboratory of Agricultural Product Processing Technology and Equipment at the College of Engineering, China Agricultural University [27]. The CVS consisted of an illumination room equipped with fluorescent lamps (J&K Photoelectronic System Co., Ltd., Shanghai, China), an industrial camera (Aca250014-gc, Basler, Ahrensburg, Germany), and a computer with MATLAB software (R2017b, The MathWorks, Inc., Natick, MA, USA). The distance between the tested samples and the camera lens was set to 15 cm. The illumination intensity of the fluorescent lamps was set to 112.

### 2.7. Texture Measurement (TM)

The dried MF samples that were processed with different processing techniques were placed on a TA-XT Texture Analyzer (Brookfield Engineering Laboratories, Inc., Middleboro, MA, USA) equipped with a P/35 probe for conducting texture profile analysis (TPA). We followed the measurement method described by Ella et al. [28] with slight modifications by setting the pre-test speed to 5.0 mm/s, test speed to 1 mm/s, post-test speed to 2 mm/s, and compression time interval between two tests to 10 s; compressing the equatorial part of the sample to a deformation of 10%; and setting the trigger point load to 20 N. The hardness of the dried MF was represented by the maximum force value obtained during the first compression. Ten dried MF samples were randomly selected for each group of experiments, and the results were averaged.

### 2.8. Intrinsic Quality Indicators

#### 2.8.1. Determination of Total Ash (TA) Content

The detection method of total ash content referred to the description in General Chapter 2302 of the 2020 Chinese Pharmacopoeia with slight modifications [1]. We accurately weighed 2.50 g of MF powder, placed it in a crucible, burned it to complete carbonization, and added 2 mL of deionized water to moisten the residue, followed by drying in a water bath. Finally, the above residues were put into a muffle furnace (SXL 1216, Jing Hong Laboratory Instrument Co., Ltd., Shanghai, China) at 550 °C for thorough ashing. Each sample was repeated three times.

#### 2.8.2. Determination of Extract Content

Traditional Chinese medicine ingredients are relatively complex, and many components are still unclear. The “extracts” index serves as a crucial parameter for monitoring the quality of medicinal materials. Following the measurement method described in General Rule 2201 of the 2020 Chinese Pharmacopoeia for determining water-soluble extractives [1]. We accurately weighed 2.00 g of the MF powder sample and added 50 mL of deionized water. We then placed the mixture in a 150 mL conical flask, allowing it to stand for 1 h. The remaining steps were carried out using the hot extraction method. The content of water-soluble extractives in the MF was calculated as a percentage based on the dry matter. Each sample was repeated three times.

#### 2.8.3. Determination of Citric Acid (CA) Content

The citric acid content is the most important quality indicator in MF and directly affects the qualification of MF products. Meanwhile, the citric acid in MF is pure and natural, which has many benefits to human health. This experiment referred to the determination method of citric acid content in MF in the 2020 Chinese Pharmacopoeia with slight modifications [1,23]. In brief, we accurately weighed 0.2 g of MF powder (passed through a no. 2 sieve in the Pharmacopoeia) and added 50 mL of deionized water. We then heated reflux for 1 h, followed by cooling and then weighing to make up for the lost weight. Subsequently, we shook well and filtered the above samples, and then the filtrate was passed through a 0.22 μm aqueous membrane (PES membrane). The obtained liquid was stored at 4 °C for further analysis.

The citric acid content was determined by using high-performance liquid chromatography (HPLC, Waters Technology Co., Ltd., Shanghai, China) equipped with a UV detector. The chromatographic column was Inertsil ODs-3, 4.6 × 250 mm (GL Sciences Inc., Shanghai, China). The column temperature was 30 °C, and the mobile phase was 0.5% ammonium dihydrogen phosphate and acetonitrile (97:3, adjusted with phosphoric acid to a pH of 3). The flow rate was 1 mL/min, with a reference sample injection volume of 10 μL and a test sample injection volume of 5 μL. The elution time was 30 min. The citric acid content was calculated based on the dried product. Each sample was repeated three times.

#### 2.8.4. Determination of Total Phenolic (TP) Content

Polyphenols play a crucial role as bioactive substances in MF. The total phenolic content in MF was determined according to the modified Folin–Ciocalteu method described by Shahidi et al. [29]. A 40 μL aliquot of MF extract was mixed with 200 μL of the Folin–Ciocalteu reagent and left at room temperature (25 °C) in the dark for 3 min. Then, 200 μL of a saturated Na_2_CO_3_ solution and 360 μL of distilled water were added to the mixture, which was then further incubated in the dark at room temperature for 30 min. The entire mixture was transferred to a 1 mL glass cuvette, and the absorbance at 760 nm was measured by using a UV-1800 spectrophotometer (Shimadzu, Kyoto, Japan). The content of total polyphenols in MF was calculated as milligrams of gallic acid equivalents per gram of dry matter (mg CAE/g DM), using quercetin as a standard. Each sample was repeated three times.

#### 2.8.5. Determination of Total Flavonoid (TF) Content

Flavonoids are another important bioactive substance in MF. The total flavonoid content in MF was determined by using the modified NaNO_2_-Al(NO_3_)_3_-NaOH spectrophotometric method described by Li et al. [30]. A 200 μL aliquot of MF extract was mixed with 60 μL of a NaNO_2_ reagent, shaken thoroughly, and left at room temperature (25 °C) for 6 min. Then, 120 μL of an Al(NO_3_)_3_ reagent was added to the mixture, shaken well, and further incubated at room temperature for 6 min. Finally, 420 μL of a NaOH reagent was added to the mixture, shaken well, and left at room temperature for 15 min. The entire mixture was transferred to a 1 mL glass cuvette, and the absorbance at 510 nm was measured. The content of total flavonoids in MF was calculated as milligrams of rutin equivalents per gram of dry matter (mg GAE/g DM). Each sample was repeated three times.

### 2.9. Total Antioxidant Capacity (TAC)

Antioxidant capacity plays a significant role in studying the pharmacological effects and health benefits of phytochemical-rich foods. The total antioxidant capacity of MF was determined based on the iron ion reduction/antioxidant capacity assay (FRAP), with slight modifications as described by Benzie et al. [31]. Briefly, 75 μL of MF extract was mixed with 75 μL of distilled water and 850 μL of the FRAP reagent, followed by thorough shaking. The mixture reacted at room temperature (25 °C) for 10 min. Subsequently, the entire solution was transferred to a 1 mL glass cuvette, and the absorbance at 590 nm was measured. The total antioxidant capacity of MF was expressed as milligrams of Trolox equivalents per gram of dry matter (mg TE/g DM). Each sample was repeated three times.

### 2.10. Statistical Analyses

The experimental data were expressed as the mean ± standard deviation of three replicates. Statistical analysis and Duncan’s multiple range test were performed by using SPSS software (version 21.0, SPSS Inc., Chicago, IL, USA) to examine the browning promotion characteristics, drying characteristics, and quality of MF under different temperature and humidity conditions. The significance level was set to 0.05.

## 3. Results and Discussion

### 3.1. Relationship between Moisture Content (MC) and Water Activity (A_W_)

**Figure 3:**
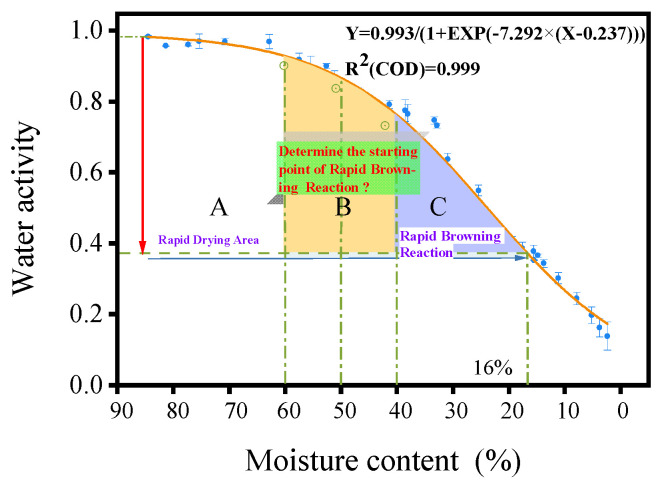
The correlation between MC and A_W_ during the drying process of PM.

A_W_ is commonly used to represent the water availability in food, which plays a decisive role in various biochemical reactions [32]. It has been found that a non-enzymatic browning reaction, mainly a Maillard reaction, occurred during the processing of PM into MF [10]. In this work, 30 groups of test data were used as fitting parameters, and the relationship between internal MC and A_W_ in PM was estimated by using SPSS software. A mathematical model was established as follows: Y = A/(B + EXP(C × (X − D))), and the mathematical expression Y = 0.993/(1 + EXP(−7.292 × (X − 0.237))) was obtained with a goodness of fit of 0.999. As shown in Figure 3. The water activity values were verified for internal MCs of 10%, 20%, and 30%, obtaining A_W_ values of 0.272, 0.419, and 0.621, respectively, which were close to the estimated values of 0.267, 0.430, and 0.606, respectively, satisfying the functional relationship between MC and A_W_. Some studies have reported that NEB would be very active when A_W_ is between 0.7 and 0.9 [33,34]. Additionally, within a certain range, NEB increases with an increase in A_W_. In the preliminary experiment, we conducted a comprehensive dehydration process on fresh PM and investigated the variation in the browning index during the processing of PM as the moisture content decreased. We found that when the moisture content of PM exceeded 65%, the browning index fluctuated within the range of 90.41 ± 0.38 to 92.73 ± 1.39, showing relatively minor changes. However, as the moisture content decreased from 65% to 60%, the browning index decreased from 90.41 ± 0.38 to 86.60 ± 1.08. At a moisture content of 50%, the browning index further decreased to 83.72 ± 0.89, and at 40%, it dropped to 63.99 ± 1.39. In the moisture content range of 60% to 40%, the browning index changed significantly. Thus, when the MC of PM was between 40% and 60%, a rapid browning reaction occurred, and the values of corresponding A_W_ were 0.761, 0.866, and 0.927. According to the 2020 Chinese Pharmacopoeia, the safe MC of MF was 16% [1] when the corresponding value of A_W_ was 0.361 in this work.

### 3.2. Suitable Moisture Transition Point for THPC-HAD Processing of MF

In order to find the optimal moisture transition point during the processing of PM into MF, which was the optimal initial moisture content for NEB, this study used THPC-HAD to dehydrate PM to different target moisture contents (TMC: 40%, 50%, and 60%). The changes in color and processing efficiency of PM were evaluated. The color parameters L*, a*, b*, Δ*E*, *C*^0^, and *BI* during processing are shown in Table 1. The results revealed that as the MC decreased from 85.89 ± 0.8% to 40%, the L* value and b* value decreased, while the a* value first increased and then decreased, and reached the maximum value when the MC was 50%. This indicated that the brightness gradually shifted from white to black, the blue-yellow value gradually shifted from yellow to blue, and the green-red value gradually shifted from green to red. The *C*^0^ value decreased from 37.97 to 12.73 (*p* < 0.05), and the fresh PM had the highest *C*^0^ value, indicating that fresh fruits had fuller and more intense colors. As the processing progressed, the color gradually darkened and the saturation decreased, which was related to the drying dehydration and browning reaction during processing. The *BI* decreased from 92.73 to 63.99 (*p* < 0.05), which showed an overall declining trend. The *BI* decreased only by 3.33% from 86.60 at 60% MC to 83.72 at 50% MC, while it decreased by 23.57% from 63.99 at 50% MC to 40% MC. That indicated that the degree of browning reaction was relatively minor when the MC decreased from 60% to 50%, but there was a more intense browning reaction when the MC decreased from 50% to 40%. In terms of processing time, under the same “staged drying” conditions, it took 14 h, 17 h, and 23 h to dry the PM to 60%, 50%, and 40% MC, respectively. The corresponding comprehensive processing times were 55.94 h, 52.89 h, and 50.56 h, respectively. Compared with the comprehensive processing time at 60% MC, the decrease in processing time at 50% and 40% was 5.45% and 9.62%, respectively. A lower MC would lead to a shorter *BI*, higher drying efficiency, lower energy consumption, and shorter comprehensive processing time. But it may result in the unsatisfactory quality of the final dried MF product due to an insufficient browning reaction during the transition phase. Therefore, comprehensively considering the drying time, conversion efficiency, and cost, this study selected 50% MC as the optimal moisture transition point for the THPC-HAD “staged drying” processing strategy.

### 3.3. Effect of Temperature and Humidity Conditions on Drying Characteristics of MF

**Figure 4:**
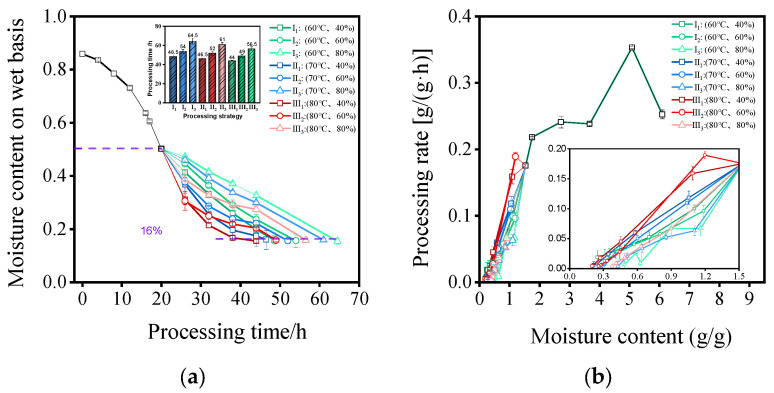
(**a**) Moisture content on wet basis curves and processing time of MF under different temperature and humidity conditions. (**b**) Processing rate curves of MF under different temperature and humidity conditions.

The impact of different processing strategies on the processing characteristics of MF is shown in Figure 4a,b. It can be observed from Figure 4a that different temperature and humidity conditions had a significant effect on the drying kinetics. As the processing temperature increased and humidity decreased, the total processing time (TPT) gradually decreased. This result is consistent with the conclusions obtained by Ju et al. in their research on the temperature and humidity control drying (THCD) of carrots [35] and Geng et al. in their research on infrared drying combined with HAD of sea buckthorn [36]. When the processing temperature was constant at 60 °C, the average TPT was 55.67 h. However, increasing the relative humidity from 40% to 60% and 80%, the TPT increased by 11.34% and 32.99%, respectively (*p* < 0.05). When the processing temperature was constant at 70 °C, the average TPT was 53.17 h, and the TPT increased by 11.83% and 31.18% when increasing the relative humidity from 40% to 60% and 80%, respectively (*p* < 0.05). When the processing temperature was constant at 80 °C, the average TPT was 49.83 h, and the TPT increased by 11.36 and 28.41% when increasing the relative humidity from 40% to 60% and 80%, respectively (*p* < 0.05). With a constant processing relative humidity of 40%, the average TPT was 46.33 h. However, elevating the temperature from 60 °C to 70 °C and 80 °C led to a reduction in TPT by 4.12% and 9.28%, respectively (*p* < 0.05). Under a constant processing relative humidity of 60%, the average TPT was 51.37 h. Increasing the temperature from 60 °C to 70 °C and 80 °C led to a TPT reduction of 3.70% and 9.26%, respectively (*p* < 0.05). Similarly, at a constant processing relative humidity of 80%, the average TPT was 60.67 h. An increase in temperature from 60 °C to 70 °C and 80 °C was associated with a TPT decrease of 5.43% and 12.40%, respectively (*p* < 0.05). When the processing conditions were set as III_1_ (80 °C, 40% RH), the shortest TPT was 44 h, followed by II_1_ (70 °C, 40% RH), I_1_ (60 °C, 40% RH), and II_2_ (80 °C, 60% RH), with a TPT of 46.50 h, 48.50 h, and 49.00 h, respectively. The longest TPT was 64.50 h for process condition I_3_ (60 °C, 80% RH). The higher the processing temperature, the greater the temperature saturation difference of the drying medium, and the more water vapor required to reach saturation, which made moisture migration inside the material easier and reduced the required time. However, appropriately increasing the processing relative humidity can promote the browning reaction inside the material. This was beneficial for improving the quality of materials, such as MF, *Polygonatum sibiricum* Red, and black garlic, that require browning [37].

Figure 4b reflects the variation in the drying efficiency of MF under different processing conditions concerning the MC on a dry basis. It can be observed that, during the processing of PM into dried MF, the drying curve contained three stages: acceleration, deceleration, and constant speed. The drying rate showed an overall increasing and then decreasing trend with the decrease in MC. In the early stage of drying, the drying rate increased rapidly to reach the maximum rate and then decreased quickly. In the middle stage of drying, the drying rate remained at a relatively stable level with a relatively fast decrease. In the final stage of drying, the drying rate decreased rapidly at a slower overall rate. In the initial drying stage, the internal temperature of PM gradually rose, accompanied by a higher internal MC. This condition resulted in a substantial pressure gradient of water vapor between the surface and the medium, facilitating the efficient removal of moisture. In the middle stage of drying, with a decrease in the MC of PM, the migration of moisture became more challenging, resulting in a gradual reduction in the rate of moisture decrease. In the late drying stage, the formation of a relatively rigid shell on the surface hindered the outward diffusion of moisture. Furthermore, the drying rate experienced a notable decline with increasing humidity at identical temperatures (60 °C, 70 °C, and 80 °C). Notably, processing conditions I_3_ (60 °C, 80% RH) and II_3_ (70 °C, 80% RH) exhibited the lowest drying rates. However, in the middle and late drying stages, the drying rate of processing condition III_2_ (80 °C, 60% RH) surpassed that of processing condition III_1_ (80 °C, 40% RH). This phenomenon may be attributed to the higher air enthalpy value at a relative humidity of 60%, providing more energy for PM and facilitating moisture diffusion from the inside to the outside. Similar conclusions were drawn by Yu et al. in their research on the drying of carrots using temperature and humidity control [38].

### 3.4. Effect of Temperature and Humidity Conditions on BPC and TPA of MF

Colors and textures are two key indicators that could promote consumer purchasing motivations [39]. Table 2 shows the impact of different processing methods on the appearance color and texture indicators of MF. Notably, an increase in temperature corresponded to a gradual decrease in the *BI*, which was considerably different from the initial value and indicated a heightened degree of browning reaction. However, the *BI* of process III_1_ (80 °C, 40% RH) increased, mainly due to the relatively high processing temperature and low relative humidity, which greatly shortened the overall processing time and led to an insufficient browning reaction. Additionally, under different temperature conditions, the relative humidity changed in different patterns. Under the conditions of 60 °C and 80 °C, the degree of browning increased with the rise in the relative humidity of the drying medium. However, under the condition of 70 °C, the degree of browning first increased and then decreased. On the one hand, it is possibly due to inherent differences between the materials. On the other hand, under the condition of 70 °C, higher relative humidity hindered the internal moisture migration in PM, thereby inhibiting the occurrence of browning reactions and leading to an increase in the *BI*.

Texture characteristics play a significant role in the external quality evaluation of MF, including hardness, elasticity, cohesiveness, chewiness, adhesiveness, and resilience parameters [40,41]. Table 2 presents the results of the texture parameter analysis of MF under different processing conditions. There were significant differences (*p* < 0.05) in the hardness, adhesiveness, and chewiness of MF plums under different processing conditions, and these parameters of processes II_1_ (70 °C, 40% RH) and III_1_ (80 °C, 40% RH) exhibited significantly higher values compared to other processed samples. This may be attributed to the higher temperature and lower relative humidity of the drying medium, resulting in the formation of a crust during the processing of MF [42]. Moreover, the hardness, adhesiveness, and chewiness of the samples showed an upward trend with increasing temperature. Under the same temperature condition, an increase in the relative humidity of the drying medium led to a significant decrease in hardness, adhesiveness, and chewiness. There was no apparent pattern observed for elasticity, cohesiveness, and resilience among MF under different processing methods. The elasticity values of dried MF ranged from 0.56 to 0.88, and processes II_2_ (70 °C, 60% RH) and III_2_ (80 °C, 60% RH) exhibited better elasticity, both above 0.8. The cohesiveness values of dried MF ranged from 0.50 to 0.74, and processes I_3_ (60 °C, 80% RH), III_2_ (80 °C, 60% RH), and III_3_ (80 °C, 80% RH) showed relatively good cohesiveness, with all being below 0.6. The resilience values of dried MF ranged from 0.19 to 0.38, and process III_2_ (80 °C, 60% RH) had the best resilience.

### 3.5. Effect of Temperature and Humidity Conditions on TA, Extracts, and CA in MF

**Figure 5:**
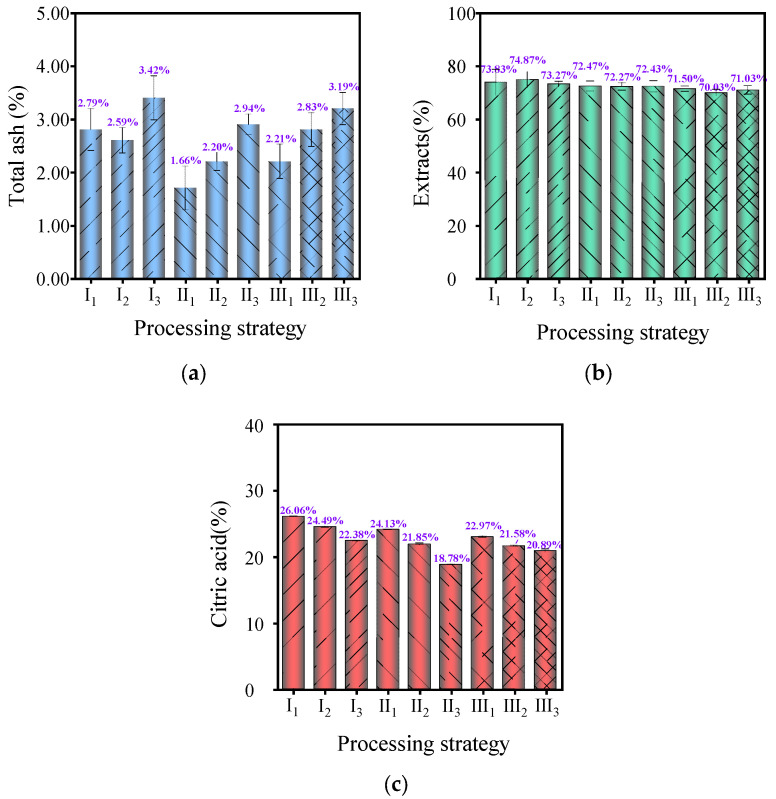
(**a**) The total ash (TA) content of MF in different temperature and humidity conditions; (**b**) The extracts content of MF in different temperature and humidity conditions; and (**c**) the citric acid (CA) content of MF in different temperature and humidity conditions.

TA content, extracts, and CA are important indicators for evaluating the intrinsic quality of MF. Among them, TA content is a crucial parameter for evaluating the quality of medicinal materials. It reflects the extent of adulteration and contamination in medicinal materials and the growth quality and processing technology of MF. According to Figure 5a, the TA content of dry MF samples under different processing conditions ranged from 1.66 ± 0.41% to 3.42 ± 0.42%, all of which met the requirement in the 2020 Chinese Pharmacopoeia of not exceeding 5.0% [1]. This indicated that the PM raw materials had a good growth quality and were subjected to effective processing technologies, but there were certain differences between samples (*p* < 0.05). When the temperature was constant, the TA content exhibited an upward trend with an increase in humidity. This may be attributed to the reactions that occurred between moisture and inorganic salts with minerals under relatively high relative humidity conditions, which led to changes in the composition of ash [43] and increased the proportion of TA to some extent.

Extracts usually serve as quality control standards for Chinese medicinal materials and are another important indicator for measuring the intrinsic quality of MF. As depicted in Figure 5b, the content of extracts in dried MF samples under different processing conditions ranged from 70.03 ± 1.29% to 74.87 ± 2.89%, which was significantly higher than the requirement of not less than 24.0% in the 2020 Chinese Pharmacopoeia [1]. Mechanized processing can significantly increase the content of extracts in MF. At the same time, the content of extracts slightly decreased with the increase in temperature, which may be attributed to oxidation reactions occurring under high-temperature conditions [44].

CA in MF has various effects such as strong antioxidant, anti-inflammatory, antibacterial, and antithrombotic properties. As illustrated in Figure 5c, the content of CA in dried MF samples under different processing conditions varied from 18.78 ± 0.06% to 26.06 ± 0.05% and showed significant differences (*p* < 0.05). Generally, the content of CA decreased with the increase in temperature and relative humidity of the drying medium. Notably, the samples under process condition I_1_ (60 °C, 40% RH) had the highest content of CA, reaching 26.06%, while the samples under process condition II_3_ (70 °C, 80% RH) had the lowest content of CA, which was 18.78%.This may be because the activities of oxalic acid enzyme and isocitric acid enzyme increased with the increase in temperature, which led to an accelerated degradation of citric acid. In addition, increased humidity extended the processing time, which can also contribute to citric acid degradation [45,46].

### 3.6. Effect of Temperature and Humidity Conditions on TP, TF, and TAC in MF

**Figure 6:**
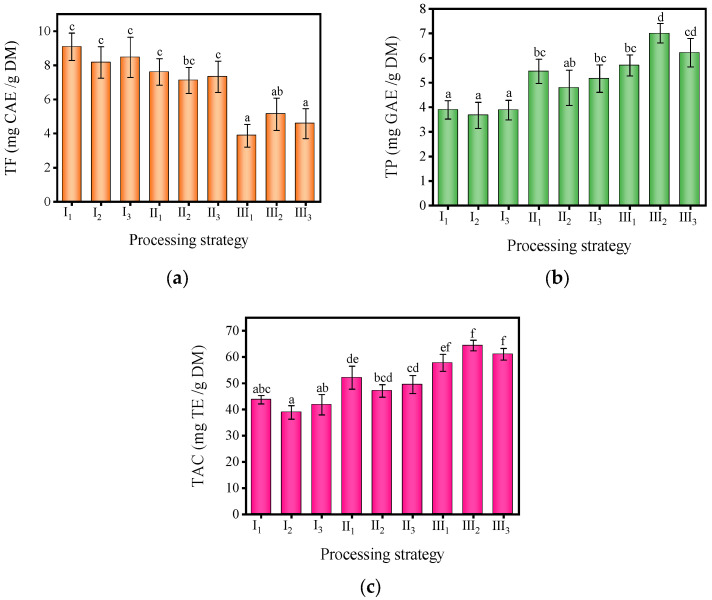
The content of (**a**) total phenolic total ash (TP), (**b**) total flavonoid (TF), and (**c**) total antioxidant capacity (TAC) of MF in different temperature and humidity conditions. (The different lowercase letters in the same picture reveal significant differences (*p* < 0.05) according to the Duncan test).

MF contains rich polyphenols and flavonoids, which offer various health benefits [5]. With the advancement of technology and medicine, people are increasingly aware of and personally experiencing the numerous health benefits associated with polyphenols and flavonoids. It is of great value to know the contents of TP and TF in MF under various processing conditions. Figure 6 illustrates the significant impact of diverse processing conditions on the content of TF, TP, and TAC in MF (*p* < 0.05). The TF content in the nine groups of MF samples exhibited a range between 3.87 ± 0.66 and 9.09 ± 0.81 mg CAE/g DM. The samples of process I_1_ (60 °C, 40% RH) had the highest TF content, while the samples of process III_1_ (80 °C, 40% RH) had the lowest TF content. The TF content decreased gradually with the increase in processing temperature, which may be due to the accelerated degradation rate of TF under high-temperature conditions [47,48]. The influence of relative humidity on the TF content was relatively special. Under temperature conditions of 60 °C and 70 °C, the TF content decreased first and then increased with the increase in relative humidity, and the increase amplitude was less than the decrease amplitude. However, under a temperature condition of 80 °C, this pattern changed. The TF content increased first and then decreased with the increase in relative humidity, and the maximum value was reached at 60% relative humidity.

Different processing conditions made a significant impact on the TP content in MF (*p* < 0.05). It can be seen from the green part in Figure 6b that the variation range of TP content in nine groups of MF samples exhibited a range between 3.67 ± 0.53 and 7.01 ± 0.39 mg GAE/g DM. The samples of process I_2_ (60 °C, 60% RH) had the lowest TP content, while the samples of process III_2_ (80 °C, 60% RH) had the highest TP content. It was worth noting that the TP content increased with the increase in temperature, which was different from the variation pattern of TF and the content of TP in various fruits, vegetables, and medicinal materials, which tended to decrease with the increase in temperature [49]. This was mainly because the high temperature promoted the release of phenolic substances bound with the cell wall in plant cells [50]. At the same time, the modification in TP content had similarities with the change in TF content. For example, under the same temperature, the TP content decreased first and then increased with the increase in relative humidity when the condition was 60 °C or 70 °C. However, at 80 °C, the TP content increased first and then decreased with the increase in relative humidity. This may be due to the fact that at relatively low temperatures (60 °C and 70 °C), higher relative humidity can effectively inhibit the degradation of TP. However, under high-temperature conditions (80 °C), increasing relative humidity leads to the certain degradation of TF due to the extension of processing time [51].

The purple part in Figure 6c illustrates the impact of different processing conditions on the TAC of MF. The iron-reducing antioxidant capacity of the nine groups of MF samples ranged from 38.86 ± 2.60 to 64.38 ± 2.04 mg TE/g DM. The antioxidant capacity of process III_2_ (80 °C, 60% RH) MF samples was the strongest, while that of process I_2_ (60 °C, 40% RH) MF samples was the weakest. This was consistent with the findings of some researchers that lower processing temperatures can prolong the drying time of samples and cause a decrease in TAC [52]. The TAC of the samples increased with the increase in temperature, and its change image was consistent with that of TP. Therefore, the TAC modification of MF was closely related to the release of high antioxidant phenolic substances [53]. At the same time, from the perspectives of I_2_ (60 °C, 60% RH) and II_1_ (70 °C, 40% RH), the TAC was also affected by the TF content. Additionally, previous studies on the color changes of samples had confirmed that violent browning reactions occurred during its processing, and the degree of browning became more severe with increasing temperature. Therefore, high-temperature conditions led to the generation of more Maillard reaction products with antioxidant activity in MF [54], which may be the reason for improving its TAC.

## 4. Conclusions

This study took MF with medicinal and edible uses as the research object and developed a multi-stage variable rapid processing strategy for browning-type materials based on THPC-HAD. In the initial stage, a high-temperature and medium-humidity (80 °C, 40% RH) process condition was used to ensure a higher drying rate and lower crust formation rate in the early drying stage of PM and ensure that the PM is quickly dried to the optimal starting moisture content (50% MC) for RBR. Then, a high-temperature and high-humidity (80 °C, 60% RH) process condition was applied to promote the browning reaction of PM for 24 h, allowing for sufficient internal reaction and conversion while further reducing the MC. Finally, the low-temperature dehumidification process (60 °C, dehumidification mode) was adopted to ensure the drying rate in the later stage of processing without affecting the quality of the MF. Through experimental verification, the proposed process method could reduce the processing cycle by 23.99% compared to other approaches; in comparison to the traditional smoking process, it could save an average energy of 1.55 kWh during the processing of 1 kg MF. Meanwhile, the method can not only improve drying uniformity, promote the browning reaction of MF, and increase the total phenolic content and total antioxidant capacity of MF but also better control the content of ash, leachate, and citric acid at a relatively high standard. This research will contribute to promoting the large-scale industrial production of browning agricultural products.

## Figures and Tables

**Table 1:** Color change parameters and images during PM processing.

Processing Time/h	0	14	17	23
Image	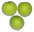	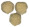	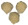	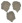
MC	85.89 ± 0.8%	60%	50%	40%
L*	54.41 ± 1.59 ^a^	38.89 ± 0.96 ^bc^	36.83 ± 0.61 ^c^	28.58 ± 0.81 ^d^
a*	−7.57 ± 0.79 ^a^	6.81 ± 0.59 ^b^	7.21 ± 0.62 ^b^	6.64 ± 0.34 ^b^
b*	37.20 ± 1.34 ^a^	20.79 ± 1.05 ^bc^	19.47 ± 0.93 ^c^	10.86 ± 1.00 ^d^
Δ*E*	-	26.79 ± 0.92 ^a^	29.03 ± 0.56 ^b^	39.54 ± 1.11 ^c^
*C* ^0^	37.97 ± 1.27 ^a^	21.88 ± 1.18 ^bc^	20.76 ± 1.07 ^c^	12.73 ± 1.00 ^d^
*BI*	92.73 ± 1.39 ^a^	86.60 ± 1.08 ^b^	83.72 ± 0.89 ^c^	63.99 ± 1.39 ^d^
Average Processing Time at 60 °C/h	55.89	45.83	38.67	29.17
Average Processing Time at 70 °C/h	53.28	42.17	36.17	27.50
Average Processing Time at 80 °C/h	50.22	37.83	32.83	26.00
Average	53.13	41.94	35.89	27.56

Note: the different letters in the same row reveal significant differences (*p* < 0.05) according to the Duncan test.

**Table 2:** *BI* and texture profile analysis (TPA) of different temperature and humidity conditions.

Processing Condition	*BI*	TPA					
		Hardness (N)	Springiness (mm)	Cohesiveness	Gumminess (N)	Chewiness (mJ)	Resilience
I_1_	44.83 ± 1.52 ^f^	68.03 ± 3.97 ^c^	0.60 ± 0.04 ^a^	0.69 ± 0.05 ^cd^	46.81 ± 0.11 ^c^	28.18 ± 1.71 ^bc^	0.27 ± 0.03 ^ab^
I_2_	41.26 ± 1.50 ^ef^	44.70 ± 3.81 ^b^	0.76 ± 0.04 ^cd^	0.62 ± 0.05 ^abc^	27.51 ± 0.66 ^b^	20.97 ± 1.46 ^b^	0.26 ± 0.03 ^ab^
I_3_	38.40 ± 1.79 ^de^	27.80 ± 2.96 ^a^	0.76 ± 0.04 ^cd^	0.56 ± 0.04 ^a^	15.56 ± 0.87 ^a^	11.82 ± 1.16 ^a^	0.23 ± 0.04 ^a^
II_1_	34.29 ± 1.27 ^cd^	150.68 ± 8.65 ^e^	0.78 ± 0.04 ^cde^	0.66 ± 0.04 ^cd^	99.99 ± 11.83 ^d^	78.00 ± 12.55 ^e^	0.30 ± 0.02 ^bc^
II_2_	31.03 ± 1.38 ^c^	70.12 ± 3.87 ^c^	0.71 ± 0.05 ^bc^	0.65 ± 0.03 ^bcd^	45.50 ± 3.80 ^c^	32.04 ± 3.54 ^c^	0.27 ± 0.03 ^ab^
II_3_	34.75 ± 2.51 ^cd^	42.08 ± 4.72 ^b^	0.84 ± 0.04 ^e^	0.65 ± 0.04 ^bcd^	27.16 ± 2.43 ^b^	22.81 ± 3.10 b^c^	0.26 ± 0.02 ^ab^
III_1_	38.64 ± 4.09 ^de^	189.45 ± 9.60 ^f^	0.67 ± 0.04 ^ab^	0.69 ± 0.04 ^cd^	129.78 ± 3.48 ^e^	86.45 ± 6.04 ^e^	0.31 ± 0.02 ^bc^
III_2_	23.65 ± 2.12 ^b^	93.99 ± 6.50 ^d^	0.82 ± 0.04 ^de^	0.55 ± 0.05 ^a^	51.26 ± 2.92 ^c^	42.07 ± 3.81 ^d^	0.35 ± 0.03 ^c^
III_3_	12.72 ± 2.71 ^a^	50.54 ± 5.42 ^b^	0.75 ± 0.04 ^cd^	0.57 ± 0.05 ^ab^	28.81 ± 1.01 ^b^	21.38 ± 1.07 ^b^	0.27 ± 0.03 ^ab^

Note: The different letters in the same column reveal significant differences (*p* < 0.05) according to the Duncan test.

## Data Availability

The data presented in this study are available on request from the corresponding author. The data are not publicly available due to privacy.
